# Immune Cells Characteristics and Their Prognostic Effects in Exertional Heatstroke Patients: A Retrospective Cohort Study

**DOI:** 10.3389/fmed.2022.867774

**Published:** 2022-04-01

**Authors:** Jingjing Ji, Peng Su, Wenyi Lin, Leifang Ouyang, Conglin Wang, Jinxin Jia, Zheying Liu, Zhifeng Liu

**Affiliations:** ^1^Department of Critical Care Medicine, General Hospital of Southern Theater Command of PLA, Guangzhou, China; ^2^Department of Medical Administration, General Hospital of Southern Theater Command of PLA, Guangzhou, China; ^3^Department of Obstetrics and Gynecology, General Hospital of Southern Theater Command of PLA, Guangzhou, China

**Keywords:** immune, neutrophil, lymphocyte, prognosis, exertional heatstroke

## Abstract

**Background:**

Exertional heatstroke (EHS) remains a major problem for those who take strenuous physical activity. Inflammation and immune dysfunction were thought to be crucial to the pathophysiological process of heatstroke. The present study was aimed to investigate the dynamic changes of the immune cells in patients with EHS and determine their prognostic effects to provide the clinical evidence of the above process.

**Methods:**

This single-center retrospective cohort study collected all patients with EHS admitted to the intensive care unit (ICU) of the General Hospital of Southern Theater Command of PLA from October 2008 to May 2019. The dynamic changes of the main immune cell count and ratio were collected, including white blood cell (WBC), neutrophil, monocyte, and lymphocyte. The neutrophil-to-lymphocyte ratios (NLR) were calculated by the neutrophil count/lymphocyte count × 100%. The main outcome was 90-day mortality.

**Results:**

A total of 189 patients were enrolled. For survivors, after 24 h, the WBC and neutrophil counts began to decrease, and they were back to normal in 72 h. In addition, the lymphocyte counts were within normal limits all the time. For non-survivors, the WBC and neutrophil counts were continuous over the normal range, while the lymphocyte count and the ratio began to decrease after 24 h and were continuously low in the following days. Receiver operating characteristic (ROC) curves analysis showed that increased neutrophils and decreased lymphocytes were associated with the poor prognosis of the patients. A prediction model based on immune cell counts and ratios was constructed, and the lymphocyte count was accounted for the maximum weight.

**Conclusions:**

In patients with EHS, increased neutrophils and decreased lymphocytes were associated with the poor prognosis. The lymphocyte count at 72 h after admission was the most important prognostic factor.

## Introduction

With the development of industrialization and urbanization, the incidences of heart-related diseases are increasing, including hyperthermia, heat edema, heat cramps, heat syncope, heat exhaustion, and heat stroke (1). Heatstroke is the most life-threatening condition, which is diagnosed as a significant elevation of core body temperature with central nervous system dysfunction, such as combativeness, delirium, seizures, and coma (2). According to its cause of onset, heatstroke has been categorized as classical heatstroke (CHS) and exertional heat stroke (EHS). The EHS is associated with strenuous physical activity in hot environments; the heat accumulation is mainly attributed to the excessive production of metabolic (3). DuBose et al. found that exercise could increase the WBC, neutrophil, monocyte, and lymphocyte count in circulation. For the exertional heat injury cases, those not meeting the heatstroke criteria, they showed increased WBC, neutrophil, and monocyte counts and decreased T lymphocyte counts (4). For now, the pathophysiological process of heatstroke is regarded as a “sepsis-like” process (5). Thus, inflammation and immune dysfunction are thought to be crucial for the development of heatstroke. Under heatstroke conditions, the damage-associated molecular patterns (DAMPs) were increased in circulation and further activate the immune cell response and inflammatory cascade. Increased concentrations of inflammatory cytokines, including interleukin-6 and tumor necrosis factor, were also observed in a clinical study on heatstroke (6). In addition, the interleukin-8, the main chemotactic clue of neutrophils, was also increased (6). An animal experiment found that inhibition of the molecule in the inflammatory signaling pathway could decrease the inflammatory response and improve survival rates in heatstroke-induced rats (7).

An increased core temperature, the main characteristic of EHS, is thought to be the “motor” for the subsequent pathophysiological process of heatstroke, and the activated inflammatory response and immune dysfunction may contribute to the subsequent multiorgan dysfunction. However, for those patients with heatstroke, the immune cell characteristics and their prognostic effect are still unclear. This study retrospectively collected the immune cell changes, and prognostic data of patients with EHS heatstroke admitted to intensive care unit (ICU) from one center in China during a 10-year period, aimed to investigate the dynamic changes of the immune cells, and determining their prognostic effects on patients with EHS.

## Methods

### Settings

Data were collected from a total of 189 patients with EHS from October 2008 to May 2019 in the ICU of the General Hospital of Southern Theater Command of PLA in China. This study was approved by the Research Ethics Commission of General Hospital of Southern Theater Command of PLA [HE2019-8]. The requirement for informed consent was waived by the Ethics Commission.

### Participants

The inclusion criteria of patients with EHS in the present study were as follows (8): (1) with a history of strenuous activity, with or without exposure to hot and high humid weather; (2) concurrent hyperthermia (central temperature above 40°C) and neurological dysfunction, including delirium, cognitive disorders, or disturbed consciousness. The exclusion criteria: (1) existing irreversible underlying diseases affecting mortality; (2) age <18 years old; (3) pregnancy or breastfeeding. All the patients received standard treatment, including cooling, organ support, and symptomatic treatment.

### Research Procedure

The characteristics and organ function parameters and a 90-day outcome of enrolled patients were collected. The dynamic changes of the immune cell count and the ratio, including WBC, neutrophil, monocyte, lymphocyte, were collected. The time points include admission, 24 h, 48 h, and 72 h after admission. The NLR were calculated by a neutrophil count/lymphocyte count ×100%. The main outcome was 90-day mortality.

### Statistical Analysis

The continuous variables were expressed as the median and an interquartile range and were analyzed by Wilcoxon rank-sum test since most continuous variables did not show a Gaussian distribution. The ROC curves and the single-factor Cox proportional hazards model were used to evaluate the effects of immune cells on a 90-day mortality in patients with heatstroke. The odds ratio (OR) and 95% CI levels (95% CI) were presented. The 14-/28-/90-day overall survival (OS) probabilities were estimated using the nomogram. The factors used for establishing prediction model was the most significant factors based the single factor cox proportional hazards models. Concordance index (C-index) calculated by bootstrapping was used to evaluate discriminative ability. Calibration plots were used to evaluate calibrating ability. Statistical analysis was performed using the R language Version 3.4.0. The *P*-values (two-tailed) below 0.05 were considered statistically significant.

## Results

### Dynamic Changes of Immune Cells in Patients With EHS: Comparison of Survivors and Non-survivors

Data from 189 patients with exertional heatstroke were collected, and all the patients were male, with a median age of 21, without any underlying disease before the heatstroke onset. During the hospitalization, 69 of them showed acute liver injury, 82 with acute kidney injury, and 85 with rhabdomyolysis. The final causes of death included direct brain injury, early shock, disseminated intravascular coagulation (DIC), septic shock due to later infection, and multiple organ failure. The dynamic change of immune cell counts and ratios in patients with EHS are shown in Tables 1, 2. At admission, most patients showed increased WBC counts, neutrophil count, and ratio. The lymphocyte and monocyte counts were within a normal range. For survivors, after 24 h, the WBC and neutrophil counts began to decrease, and they were back to normal in 72 h. Although the lymphocyte ratio was a bit lower than the normal range, the absolute count of lymphocytes was within the normal range all the time. For non-survivors, the WBC and neutrophil counts were continuously over the normal range. The lymphocyte count and the ratio began to decrease after 24 h and were continuously low in the following days. The NLR was a new biomarker applied in prognosis' evaluation on cancer and sepsis. Our results also showed increased NLR in the acute phase of most patients with EHS. Moreover, surviving patients with EHS showed decreased NLR after 24 h, and the NLR in non-survivors was continuously over 10 (Table 2).

**Table 1:** The dynamic change of immune cell counts in patients with exertional heatstroke (EHS).

	**Overall (*N* = 189)**	**Survivor (*N* = 166)**	**Non-survivor (*N* = 23)**	***P*-value**
**WBC**				
Ad	11.36 [8.73, 14.60]	11.44 [8.91, 14.51]	10.44 [8.07, 15.45]	0.860
24 h	9.21 [6.79, 12.25]	9.19 [6.70, 12.14]	9.63 [8.36, 13.95]	0.122
48 h	8.13 [6.37, 10.28]	7.97 [6.36, 9.93]	10.08 [7.27, 13.95]	0.025
72 h	7.44 [5.89, 9.50]	7.16 [5.87, 8.96]	9.90 [7.50, 11.71]	0.015
**Neut**				
Ad	8.86 [6.55, 12.41]	9.01 [6.62, 12.23]	8.72 [6.30, 13.18]	0.952
24 h	6.97 [4.81, 10.27]	6.66 [4.54, 9.79]	8.26 [7.44, 12.95]	0.002
48 h	6.04 [4.24, 8.57]	5.79 [4.10, 7.77]	8.60 [6.70, 12.95]	<0.001
72 h	5.30 [4.05, 7.45]	4.93 [3.96, 7.13]	9.09 [6.12, 10.96]	0.001
**Lym**				
Ad	1.12 [0.58, 1.90]	1.13 [0.68, 1.86]	0.64 [0.32, 2.46]	0.164
24 h	1.31 [0.82, 1.83]	1.39 [1.05, 1.94]	0.34 [0.29, 0.47]	<0.001
48 h	1.30 [0.70, 1.87]	1.36 [0.96, 1.95]	0.34 [0.22, 0.41]	<0.001
72 h	1.28 [0.70, 1.72]	1.34 [0.87, 1.83]	0.33 [0.23, 0.52]	<0.001
**Mono**				
Ad	0.68 [0.38, 0.99]	0.68 [0.39, 1.00]	0.68 [0.25, 0.84]	0.45
24 h	0.51 [0.32, 0.73]	0.54 [0.35, 0.74]	0.12 [0.08, 0.53]	<0.001
48 h	0.43 [0.32, 0.61]	0.44 [0.34, 0.62]	0.29 [0.13, 0.40]	0.003
72 h	0.43 [0.32, 0.60]	0.43 [0.32, 0.59]	0.42 [0.18, 0.66]	0.644

**Table 2:** The dynamic changes of immune cell ratios and neutrophil to lymphocyte ratios (NLR) in patients with EHS.

	**Overall (*N* = 189)**	**Survivor (*N* = 166)**	**Non-survivor (*N* = 23)**	***P*-value**
**Neutrophil ratio (%)**				
Ad	84.15 [72.60, 88.02]	84.10 [71.80, 87.80]	84.20 [77.30, 91.10]	0.364
24 h	81.10 [72.27, 88.00]	79.90 [71.90, 85.90]	93.70 [90.20, 95.50]	<0.001
48 h	76.10 [65.47, 87.08]	73.50 [64.15, 83.45]	94.50 [91.57, 95.58]	<0.001
72 h	74.70 [64.03, 84.20]	72.30 [62.20, 82.05]	91.80 [85.25, 93.50]	<0.001
**Lymphocyte ratio (%)**				
Ad	8.80 [4.90, 18.50]	9.30 [5.40, 18.60]	7.70 [4.10, 16.10]	0.3
24 h	11.35 [6.50, 19.82]	12.70 [7.70, 20.90]	4.00 [2.80, 4.90]	<0.001
48 h	16.20 [7.40, 25.22]	17.75 [11.18, 26.13]	3.05 [2.03, 4.07]	<0.001
72 h	16.85 [8.20, 25.40]	18.50 [10.80, 26.45]	4.00 [2.50, 6.70]	<0.001
**Monocyte ratio (%)**				
Ad	5.90 [3.90, 7.70]	6.00 [4.00, 7.70]	5.00 [3.20, 8.80]	0.71
24 h	6.00 [4.00, 7.50]	6.20 [4.60, 7.60]	1.70 [1.00, 2.90]	<0.001
48 h	5.70 [3.98, 7.50]	6.05 [4.40, 7.53]	2.25 [1.10, 4.02]	<0.001
72 h	6.00 [4.70, 7.68]	6.10 [4.95, 7.70]	4.00 [2.95, 6.95]	0.018
**NLR**				
Ad	9.05 [3.92, 17.72]	8.97 [3.78, 16.51]	12.45 [4.99, 23.68]	0.187
24 h	5.52 [2.94, 11.37]	4.88 [2.62, 8.74]	25.66 [20.50, 35.49]	<0.001
48 h	4.88 [2.62, 12.36]	4.02 [2.49, 7.39]	28.78 [21.73, 44.78]	<0.001
72 h	4.39 [2.54, 10.07]	3.83 [2.40, 7.34]	23.00 [12.27, 36.02]	<0.001

### Sensitivity Analysis of Immune Cell Counts and Ratios for the Prognosis of Patients With EHS

**Figure 1:**
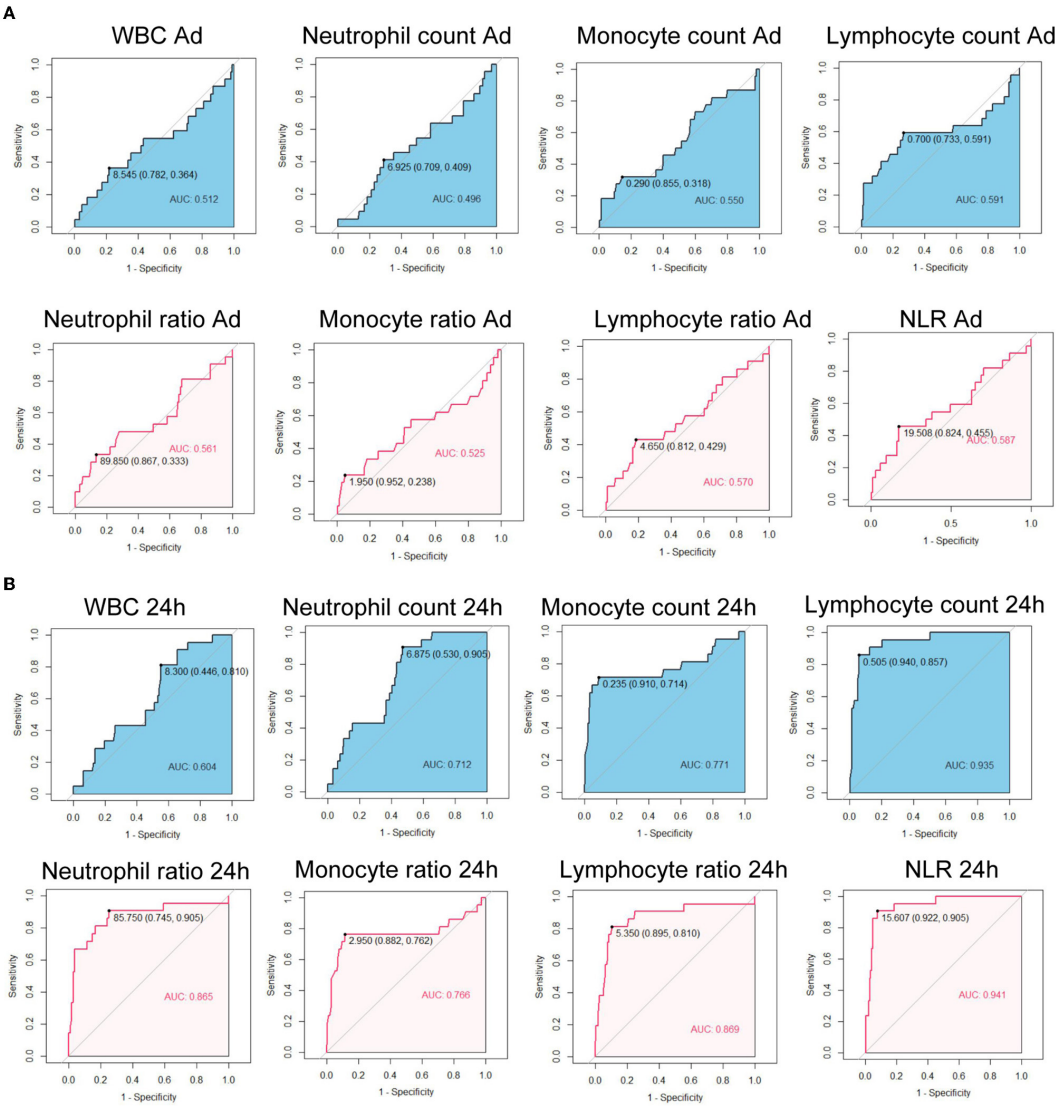
Receiver operating characteristic curves of immune cell counts and ratios at admission **(A)** and at 24 h after admission **(B)** for 90-day fatality in patients with exertional heatstroke (EHS). WBC, white blood cell; NLR, neutrophil to lymphocyte ratio; AUC, area under curve.

**Figure 2:**
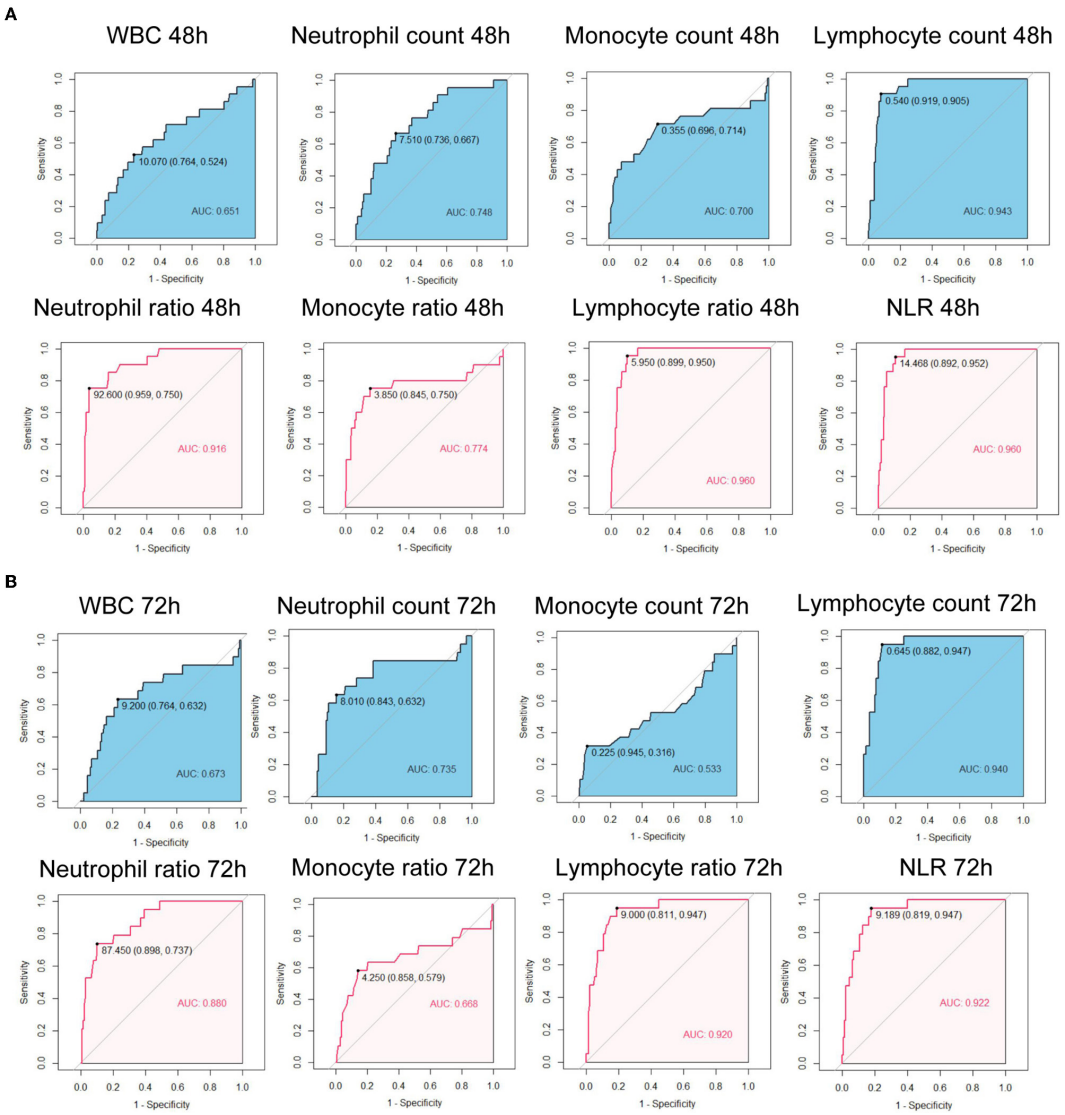
Receiver operating characteristic curves of immune cell counts and ratios at 48 h after admission **(A)** and at 72 h after admission **(B)** for 90-day fatality in patients with EHS. WBC, white blood cell; NLR, neutrophil to lymphocyte ratio; AUC, area under curve.

We further calculated the Area Under the Curves (AUCs) of immune cell counts and ratios. At admission, AUCs of all parameters were lower than.7 (Figure 1A). After 24 h, the AUC for the lymphocyte count was 0.935, with a cut-off of 0.505 × 10^6^/ml (Figure 1B). The AUC for NLR at 24 h was 0.941 with a cut-off of 15.707. At 48 h, the AUCs of the lymphocyte count, neutrophil ratio, lymphocyte ratio, and NLR were all over 0.9. The lymphocyte ratio and NLR showed the highest AUC of 0.960 (Figure 2A). At 72 h, the AUCs of NLR, the lymphocyte count, and the ratio were over 0.9. The lymphocyte count showed the highest AUC of 0.940, a cut-off of 0.645 × 10^6^/ml (Figure 2B). These results suggested that the lymphocyte count and NLR showed a higher predictive value for the prognosis of patients with EHS.

**Figure 3:**
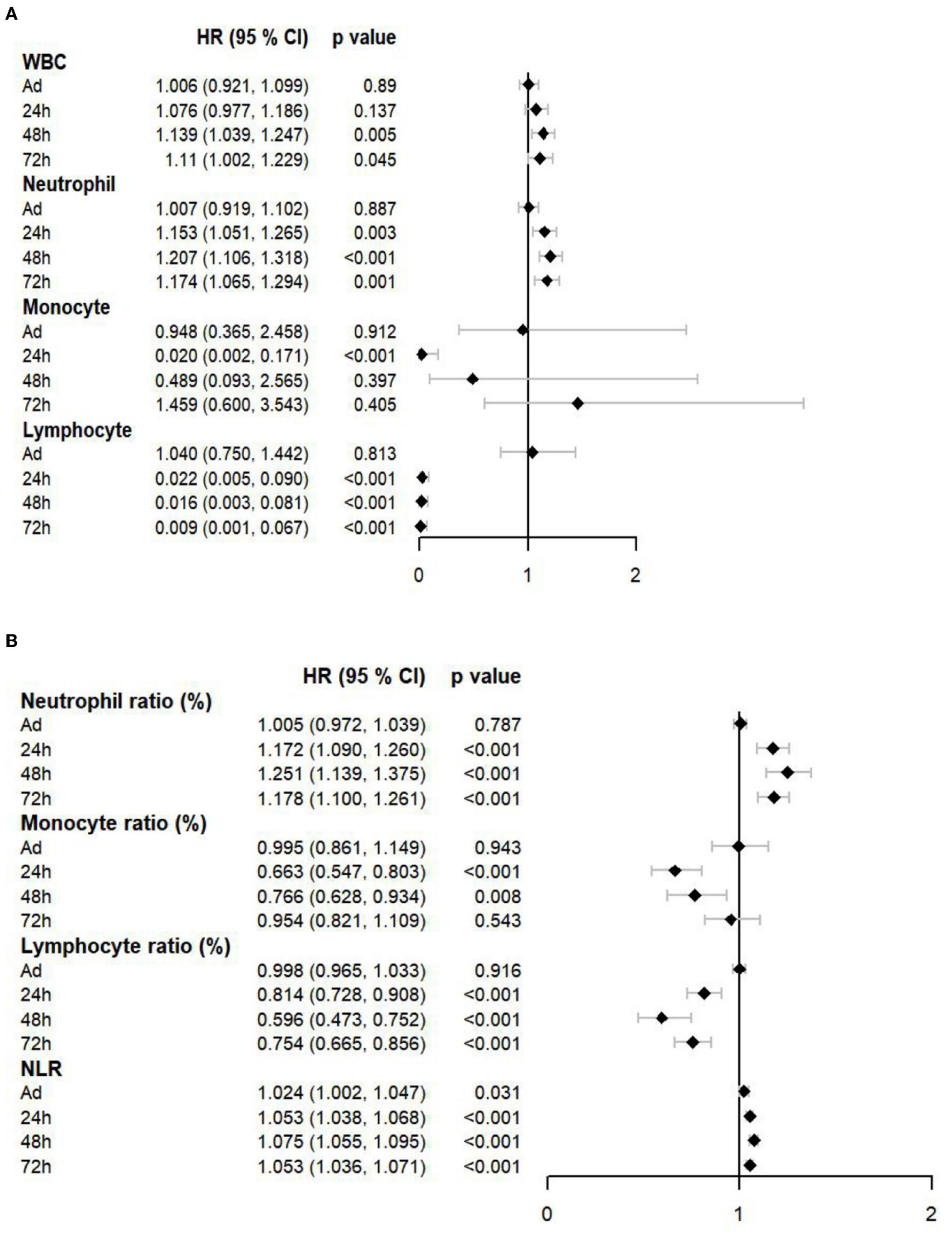
A forest plot showed the effects of immune cell counts **(A)** and a ratio **(B)** on a prognosis of patients with EHS. WBC, white blood cell; NLR, neutrophil to lymphocyte ratio; HR, hazard ratio.

The Cox proportional hazards models were also used to evaluate the effect of immune cell counts (Figure 3A) and the ratio (Figure 3B) on the prognosis of patients with EHS. Neutrophil counts and ratios at 24 h, 48 h, and 72 h showed hazard ratio (HR) over 1. At 24 h, the HR of the monocyte count was 0.020 (95% CI:0.002–0.171, *p* <0.001). The HR of the lymphocyte count and the ratio at 24 h, 48 h, and 72 h were significantly lower than 1. These results showed that increased neutrophils and decreased lymphocytes were associated with the poor prognosis of patients with EHS.

### Construction and Validation of the Prediction Model Based on Immune Cells in Patients With EHS

**Figure 4:**
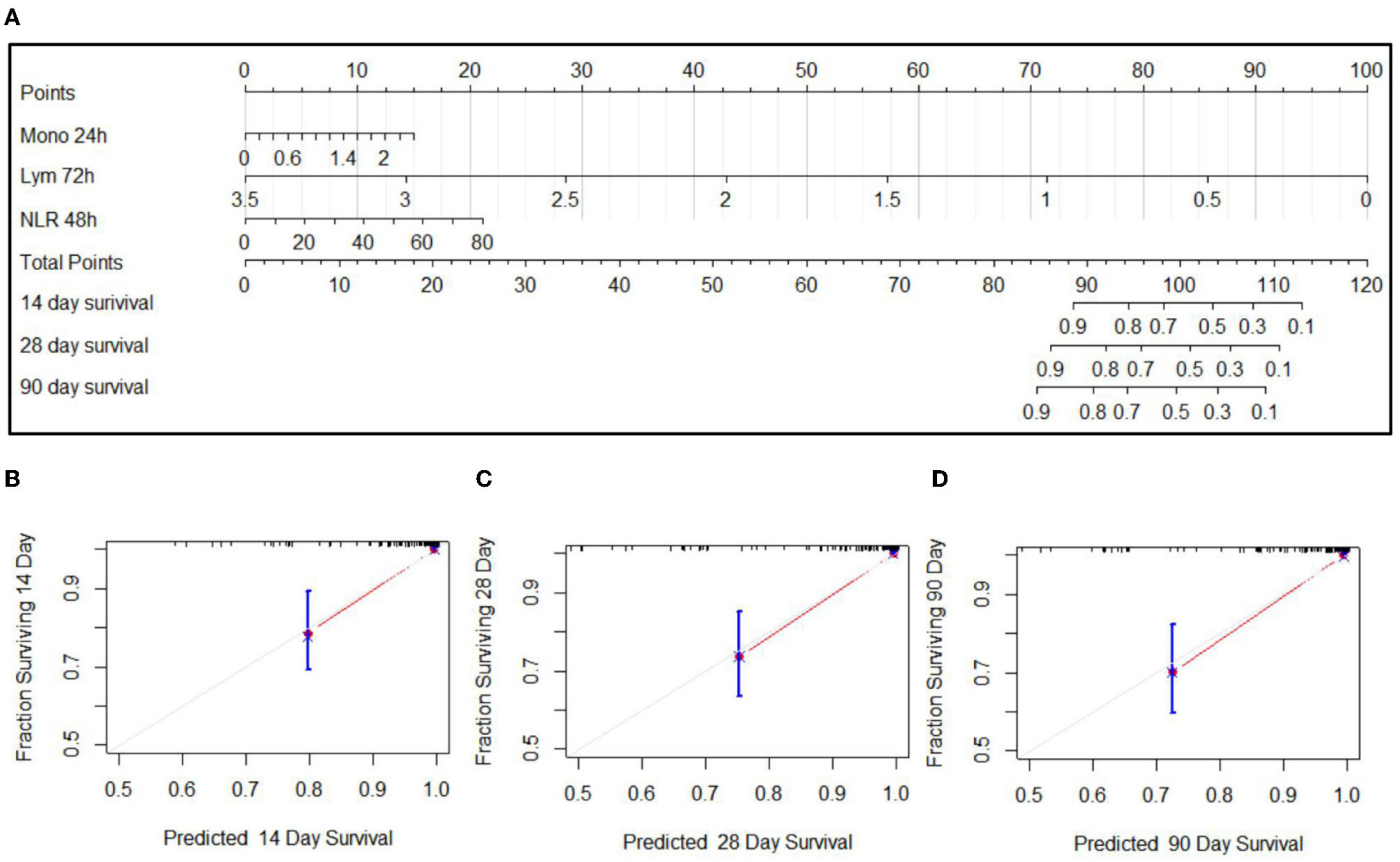
Nomogram for the predication on 14-day, 28-day, and 90-day prognoses of EHS **(A)** and calibration curves of 14-day **(B)**, 28-day **(C)**, and 90-day **(D)** of nomogram.

Since these factors were not independent, stepwise multivariate logistic regression was not suitable to screen the factors for nomogram. Based on the single-factor Cox proportional hazards models, the most significant factor of different cell types and time points was chosen, including the monocyte count at 24 h, NLR at 48 h, and the lymphocyte count at 72 h. The nomogram is shown in Figure 4A. Among the variables, the lymphocyte count took the largest proportion in this model. The decreased lymphocyte count and increased NLR were related to the increased score and a poor prognosis. The C-index was 0.926 (SE:0.022). The calibration curves of the nomogram showed high consistencies between the predicted and observed survival probability (Figures 4B–D). In summary, the nomogram had considerably discriminative and calibrating abilities.

## Discussion

The present study firstly reported that increased neutrophils and decreased lymphocytes were associated with the poor prognosis of patients with EHS. A nomogram was also constructed to predict the prognosis of those patients. The validation of the nomogram showed that it has good discriminative and calibration capabilities. Also, the lymphocyte count at 72 h after admission was the most important prognostic factor. Based on our findings, we provided a good score model to accurately predict the prognosis of patients with EHS, which provides a way to better understand the degree of severity of this critical illness and provides the clinical evidence to use immune intervention therapy in the future. The NLR has been a recently established inflammation marker that reflects systemic inflammatory response and is associated with mortality in several malignancies (9). Our results showed increased NLR in the acute phase of most patients with EHS. The NLR in surviving patients with EHS began to decrease after 24 h, and the NLR of non-survivors was continuously over 10. Increased neutrophil with or without decreased lymphocyte, or decreased lymphocyte with neutrophil in a normal limit, could result in the increased NLR.

The mechanisms related with the alterations of neutrophil number and function in heatstroke were still unclear. However, based on the existing knowledge of other inflammatory diseases, increased neutrophils counts could be related to the increased mobilization and prolonged apoptosis (10), which was thought to be related to the systemic inflammatory response and may contribute to the pathophysiological process of heatstroke. Several mechanisms were found involved in the heatstroke-induced systemic inflammation, including direct cytotoxic effects of heat, endothelial cell injury, leakage of endotoxin from the intestinal mucosa. Firstly, direct heat stress could induce the damage of a mass of cells, leading to the release of DAMPs, such as high-mobility group box 1 protein (HMGB1) (11). The DAMPs could further activate most immune cells and lead to the inflammation cascade and cytokine storm. Secondly, endothelial cells played an important role in maintaining the stability of the organ microenvironment. Endothelial cells display different toll-like reporters on their surface, which could recognize the DAMPs and activate the inflammatory signal pathway (12). In addition, the heat stress could also directly impair gut integrity, leading to leakage of endotoxin and the recruitment of immune cell populations (13). These factors contribute to the systemic inflammatory response of patients with heatstroke, resulting in increased WBC counts, neutrophil count, and ratio. Uncontrolled neutrophil responses could exacerbate the inflammatory response and lead to further organ damage (14).

For the adaptive immune system, due to the important roles lymphocytes played in the orchestration of adaptive immune responses, they greatly impacted the overall immune fitness. Our results showed that the lymphocyte count and the ratio of non-survivors began to decrease at 24 h after admission and were continuously low in the following days. In the prediction model, the lymphocyte count was accounted for the maximum weight. These results implied that continuously decreased lymphocyte was associated with a poor prognosis. The mechanism of decreased lymphocyte in heatstroke was still unclear; one of the potential mechanisms could be apoptosis. Lymphocyte populations were susceptible to apoptosis. Under the heatstroke condition, the cytokines in circulation, such as tumor necrosis factor (TNF)-α, could induce lymphocyte apoptosis (15). A published study found that, in the mice with heatstroke, the lymphocyte-deficient severe-combined immune-deficient mice showed significantly high levels of inflammatory cytokines and increased mortality rates (16). Lymphocyte counts include T cell, B cell, and NK cell. Under normal conditions, T cells were the main cell type of lymphocyte, accounting for 50–85%. A previous study found that regulatory T cells found could improve the intestinal barrier dysfunction and modulate neutrophil recruitment in a heatstroke animal model (17). In the studies from sepsis, an effective T cell immunity has been found essential to limit the frequency and severity of recrudescence of infection in humans (18). Based on these results, we believed that decreased lymphocytes might be related to the infection in patients with heatstroke and further exacerbate the prognosis, and T cells might be the main course related to the poor prognosis. Even so, the effect of B cell and NK cell should still not be ignored. B cells were responsible for the humoral immunity of the adaptive immune system by producing antibodies (19). NK cells were a type of cytotoxic lymphocyte critical to the innate immune system by eliminating an invader (20). The roles of B cells and NK cells in heatstroke are still not fully explored. Based on their known effects, decreased B cells and NK cells could also impair the immune system and increase mortality risk. Future studies are required to illustrate the exact effects of T cells, B cells, NK cells, and even their subgroups in heatstroke.

Notably, this study showed that the difference of immune cells between survivors and non-survivors was observed after 24 h after admission, even more later, not the admission. At admission, most patients showed increased neutrophil counts and ratio, suggesting that the direct effect of heat was activating and mobilizing the neutrophil. After 24 h, most patients have been cooling down to normal temperature. Heat just pulled the trigger. For survivors, all systems began to recover, including the immune cell counts and ratios. However, for non-survivors, the consistent immune disorder was related to the acute physiological alterations, including activation of endothelial, increased metabolic demands, hypoxemia, cytokines storm, *etc*. Consistent systemic inflammatory response induced the organ injury. In turn, damaged organ cells release more DAMPs and further activated the immune system, forming a vicious circle, resulting in a poor prognosis. The mechanisms of lymphocytes' changes may be similar, but the change direction is opposite. A more detailed cellular and molecular mechanisms need further basic research in the future. Hence, based on our findings of immune changes and their potential mechanisms, not only cooling but also systemic treatment was crucial for patients with heatstroke.

Although the systemic inflammatory response has been considered a crucial process in heatstroke, the evidence is still lacking. Hence, in a general guideline, most therapies were targeted in temperature management and organ function support, while immunity support was not included, at least, in the acute phase. The present study was retrospective, thus, no medicine that directly affects immunity was used. However, our results revealed that immune dysfunction was the risk factor in the prognosis and provide the clinical evidence to use immune intervention therapy in the future. In the present study, only patients with EHS were enrolled since the patients with CHS showed too many confounding factors to summarize their characteristics. The heat-induced responses of immune cells might be similar in both patients with EHS and CHS due to the similar pathophysiological response to heat, and DAMPs induced the activation and mobilization of immune cells. However, most patients with CHS are children or elder people and those patients with underlying diseases, such as cardiovascular disease; while patients with EHS are strike athletes and laborers, most of whom are young and healthy men, usually without any underlying diseases. Therefore, the immune response and the immune cell changes of patients with CHS might be more complicated than that in patients with EHS, and immune support might be more needed for them, which is also an important point in the future study.

This study also has some limitations. Firstly, this was a single-center retrospective cohort study. Most cases were in the south area of China, and the climate is dominated by high temperatures and high humidity. It was unclear whether our findings were suitable for the dry heat-induced heatstroke cases. Secondly, the present study only analyzed the immune cell counts, but other inflammatory parameters were lacking, such as inflammatory cytokines. Moreover, the cell counts cannot fully reflect the functions of different immune cells, and more functional parameters were required in the future study. Thirdly, this study only found that increased neutrophils and decreased lymphocytes were associated with the poor prognosis of patients with EHS. Further studies are required to clarify the effects of immune cell changes on organ injury and infection, even its potential cellular and molecular mechanisms.

## Conclusions

In summary, we found that increased neutrophils and decreased lymphocytes were associated with the poor prognosis in patients with EHS; the lymphocyte count at 72 h after admission was the most important prognostic factor.

## Data Availability Statement

The raw data supporting the conclusions of this article will be made available by the authors, without undue reservation.

## Ethics Statement

The studies involving human participants were reviewed and approved by Ethics Committee of General Hospital of Southern Theater Command of PLA. Written informed consent for participation was not required for this study in accordance with the national legislation and the institutional requirements.

## Author Contributions

ZhiL and JJi: study concept and design, and statistical analysis. PS,WL, LO, CW, JJia, and ZheL: data collecting. JJi, PS, and ZhiL: manuscript drafting. All authors contributed to the article and approved the submitted version.

## Funding

This work was supported by grants from the China Postdoctoral Science Foundation (2021M693956), PLA Logistics Research Project of China (18CXZ030, 17CXZ008), National Natural Science Foundation of China (No. 82072143), and Natural Science Foundation of Guangdong Province (2021A1515010170).

## Conflict of Interest

The authors declare that the research was conducted in the absence of any commercial or financial relationships that could be construed as a potential conflict of interest.

## Publisher's Note

All claims expressed in this article are solely those of the authors and do not necessarily represent those of their affiliated organizations, or those of the publisher, the editors and the reviewers. Any product that may be evaluated in this article, or claim that may be made by its manufacturer, is not guaranteed or endorsed by the publisher.
